# Population genomic screening: Ethical considerations to guide age at implementation

**DOI:** 10.3389/fgene.2022.899648

**Published:** 2022-10-04

**Authors:** Scott J. Spencer, Stephanie M. Fullerton

**Affiliations:** ^1^ Institute for Public Health Genetics, University of Washington, Seattle, WA, United States; ^2^ Department of Bioethics and Humanities *,* University of Washington, Seattle, WA, United States

**Keywords:** bundled genomic screening, population screening, principlist ethics, utilitarian ethics, implementation

## Abstract

Currently, most genetic testing involves next generation sequencing or panel testing, indicating future population-based screening will involve simultaneous testing for multiple disease risks (called here “panel testing”). Genomic screening typically focuses on single or groups of related disorders, with little utilization of panel testing. Furthermore, the optimal age for test ordering is rarely addressed in terms of whether it should coincide with the age of majority (18 years old) or after the age of majority (26 years old). We conducted an ethical analysis utilizing a hypothetical “narrow” panel test comprised of the CDC Tier 1 Genomic Applications: Familial Hypercholesterolemia (FH), increases individuals’ cardiovascular risk due to elevated low-density lipoprotein (LDL) cholesterol levels; Hereditary Breast and Ovarian Cancer (HBOC), increases lifetime risk of developing cancer; and Lynch Syndrome (LS), increases lifetime risk of developing colorectal cancer. We conducted a utilitarian analysis, on the assumption that health systems seek to maximize utility for patients. Screening at the “age of majority” is preferred for FH due to lowering FH patients’ cholesterol levels *via* statins providing high lifetime benefits and low risks. Screening “after the age of majority” is preferred for HBOC and LS due to availability of effective surveillance, the recommendation for screening activities to begin at age 26, and prophylactic interventions connected to surveillance. We also utilized a supplemental principlist-based approach that identified relevant concerns and trade-offs. Consideration of clinical, non-clinical, and family planning implications suggests narrow panel testing would be best deployed after 26 (rather than at 18) years of age.

## Introduction

Population-level genomic screening for future disease risk is one of the ultimate goals of precision medicine. (Green et al., 2015). As most genetic testing involves next generation sequencing or panel testing, it is likely that future screening will involve simultaneous testing for multiple disease risks (called here “panel testing”). (Green et al., 2013; Marshall et al., 2020). However, most decision-making about the implementation of genomic screening has focused on considerations relevant to independent conditions, with no analysis of the implications of panel testing or their relationship to the age at which such screening, ideally, would be offered.

The need for such a decision-making framework is clear. Pediatric and newborn population genomic screening have been discussed at length but there is currently only limited guidance related to genomic screening of healthy adults. (Burke et al., 2013; Committee on Bioethics Committee on Genetics ACMG Genomics SocialEthical and Legal Issues Committee, 2013; Ross et al., 2013; Clayton et al., 2014; Murray et al., 2018). Various recommendations for adult genomic screening address timing of screening, associated risk management strategies, and follow-on surveillance activities for a variety of conditions including cancers and cardiovascular disease. Age of screening takes on special significance in the context of panel testing due to interactions between the age of onset for conditions included within the panel test and the degree to which treatment or intervention is tied to the age of the patient. While it might seem straightforward to plan for offering panel testing to patients as they reach the “age of majority” (18 years old in the United States, when individuals are granted full legal and decision-making capacity; also, the age at which most can consent to medical care), various trade-offs may make implementation later in adulthood preferable. (Legal Information Institute). For example, health systems may prefer to initiate screening after 26 years of age, the age at which the U.S Department of Health & Human Services require patients to cycle off their parent’s health insurance and establish coverage on their own behalf (called here “after the age of majority”).

In anticipation of the need for systematic values-based analysis that can inform health system leaders’ decisions about the appropriate age at which to offer panel testing, we conducted an ethical analysis assuming a hypothetical “narrow” panel test comprised of just the Center for Disease Control and Prevention (CDC) Tier 1 Genomic Applications: Familial Hypercholesterolemia (FH), Hereditary Breast and Ovarian Cancer (HBOC), and Lynch Syndrome (LS). CDC Tier 1 Genomic Applications are conditions that have significant potential for positive impact on public health based on available evidence-based guidelines and recommendations. (Centers for Disease Control and Prevention, 2014). Specifically, we describe key classes of test implications (clinical, non-clinical, and family planning related) for this case and demonstrate how utilitarian and principlist frameworks might help guide decision-making about the offer of this, and potentially any, panel testing. Our analysis assumes “population” refers to a demographically representative sample of the United States. We also assumed that patients will be offered panel testing in a primary care wellness exam and have access to these services through either insurance coverage or public health initiatives. There will likely be additional Tier 1 conditions added over time and characteristics of panel testing highlighted in this analysis are intended to guide considerations of future, broader panel testing. The characteristics of this analysis are highlighted in Table 1.

**TABLE 1:** Characteristics of panel test for analysis.

Condition	Presentation	Age of onset	Screening recommendation	Treatment options
Familial Hypercholesterolemia	Prevalence of ∼1/250, that increases individuals’ cardiovascular risk primarily due to elevated low-density lipoprotein (LDL) cholesterol levels (Goldberg et al., 2011; Sjouke et al., 2015; Benn et al., 2016)	Median age of onset for coronary heart disease: 51 (IQR 42-61) (Cleveland Clinic, 2022)	Surveillance through cholesterol screening (Simon Broome Register Group, 1991; Newman et al., 2019)	Preventative intervention may provide meaningful benefit *via* lipid lowering therapy and related clinical actions (Simon Broome Register Group, 1991; Newman et al., 2019)
Hereditary Breast and Ovarian Cancer	Prevalence of ∼1/200, increased lifetime risk of developing cancer (Manickam et al., 2018)	BRCA carriers have 4% cumulative risk of breast and ovarian cancer by age 30^92^	Increased surveillance for affected individuals such as mammography or MRI (National Cancer Institute, 2021)	Prophylactic surgery such as mastectomy and/or oophorectomy is recommended after 30 years old
Lynch Syndrome	Prevalence of ∼1/300, develop colorectal and other cancers at younger ages compared to the general population (Kastrinos et al., 2008; Jasperson et al., 2010; ten Broeke et al., 2015; Oliveri et al., 2018)	Average age of CRC diagnosis between ages 30 to 50 depending on the associated gene mutation (Oliveri et al., 2018)	Individuals with LS are recommended to engage in intensive colonoscopy surveillance including annual or biennial colonoscopy surveillance beginning at 25 years old (; Degoma et al., 2016; Daly et al., 2020)	Polyps identified by screening can be resected and prophylactic surgery may be necessary such as a colectomy (Cleveland Clinic, 2022; Katz et al., 2017)

## Narrow panel test conditions

### Familial Hypercholesterolemia

FH is a common monogenic condition, with a prevalence of ∼1/250, that increases individuals’ cardiovascular risk primarily due to elevated low-density lipoprotein (LDL) cholesterol levels and independent risk associated with FH variants. (Goldberg et al., 2011; Sjouke et al., 2015; Benn et al., 2016). Individuals with untreated FH may have a 20 times higher life risk of coronary heart disease compared to the general population. (NIH). Individuals with FH also have an increased risk of experiencing a cardiovascular event earlier in life compared to individuals without FH. (Kuchenbaecker et al., 2017). In the CASCADE-FH registry in the United States the median age at FH diagnosis was 47 (IQR 31-59), the median age of initiation for LDL-lowering therapy was 39 (IQR 25-50), and median age of onset for coronary heart disease was 51 (IQR 42-61). (Cleveland Clinic, 2022).

### Hereditary breast and ovarian cancer

HBOC genetic variants confer increased lifetime risk of developing cancer. (Manickam et al., 2018). For example, BRCA1 and BRCA2 carriers experience ∼40 percent cumulative risk of breast cancer and ∼10 percent cumulative risk of ovarian cancer by the time they are 50. (Manickam et al., 2018). The prevalence of pathogenic HBOC variant carriers is ∼1/200. (Domchek et al., 2010; Dewey et al., 2016). Identification of HBOC variants allows for more intensive precancer screening practices such as magnetic resonance imaging (MRI) and for individuals to engage in chemoprevention, prophylactic risk-reducing mastectomy (RRM), and/or risk-reducing salpingo-oophorectomy (RRSO) to lower cancer risk and cancer mortality. (Hampel et al., 2008; US Preventive Services Task Force, 2019).

### Lynch syndrome

LS is the most common inherited cause of colorectal cancer (CRC), involved in ∼4% of incident cases. (Bonadona et al., 2011; Moreira et al., 2012; Ahnen et al., 2014). Individuals with LS develop cancer at younger ages compared to the general population, with an average age of CRC diagnosis between roughly 30 to 50 depending on the associated gene mutation. (Kastrinos et al., 2008; Jasperson et al., 2010; ten Broeke et al., 2015; Oliveri et al., 2018). LS is also associated with increased risk for endometrial, ovarian, and prostate cancers. (Møller et al., 2017; Dominguez-Valentin et al., 2020; ). Current guidelines recommend decennial colonoscopy surveillance for CRC beginning at 50 years old for the general population and individuals with LS are recommended to engage in intensive colonoscopy surveillance including annual or biennial colonoscopy surveillance beginning at age 25 years. ; Degoma et al., 2016; Daly et al., 2020).

## Types of test implications considered

A targeted literature review, patient interviews, and reports of expert roundtable discussions were utilized to identify implications related to panel testing for the purpose of the proposed ethical analysis. (Research on Genomics et al., 2018)^,^ (Chowdhury et al., 2013)^,^ (Khoury, 2013) This targeted review identified implications such as disease prevention, treatment, care management, patient experiences, psychosocial effects, reproductive decision-making, and other considerations. Once these implications were identified, they were characterized for implementation in the proposed ethical analysis. To simplify the ethical analysis, these implications were organized into three main categories: (1) clinical, (2) non-clinical, and (3) family planning related

Clinical implications include the extent to which a given screening test provides effective disease prevention, appropriate treatment, and/or care management. (Khoury, 2013; Research on Genomics et al., 2018). Prevention of disease includes prophylactic interventions or other recommended treatments. (NIH,; US Preventive Services Task Force, 2019; Hampel et al., 2008; ). Appropriate treatment and care management considered time sensitivity related to care, recommendations and/or evidence of an optimal age for an intervention or care pathway, and whether care management involves screening, surveillance, or clinical activities. (Bowen et al., 2012; Khoury, 2013; Research on Genomics et al., 2018).

Non-clinical implications include impacts associated with, or related to, a given screening test, including patient experiences and/or psychosocial effects. (Burke et al., 2011; Research on Genomics et al., 2018). These behavioral impacts may be difficult to quantify but require consideration because they can affect clinical utilization, surveillance adherence, and/or clinical outcomes. Family planning implications include actions or considerations related to reproductive decision-making, such as the use of carrier and/or prenatal genetic screening, cascade testing in family members, or the adjustment to treatment to enable conception. (George et al., 2015).^,^ (Lokich et al., 2014)

## Age at which to offer “narrow” panel testing: Ethical considerations

The three categories of implications were used in a two-phased ethical analysis focused on the appropriate age at which to offer a hypothetical “narrow” panel test comprised of just the CDC Tier 1 Genomic Applications (for FH, HBOC, and LS) to adult patients. First, a utilitarian framework was employed, on the assumption that health systems may similarly seek to maximize utility for patients. Next, we supplemented the analysis with a principlist-based approach that identified additional relevant concerns and trade-offs. In both analyses we consider the offer of panel testing at either the “Age of Majority” (i.e., 18 years old) or “After the Age of Majority” (i.e. 26 years or older).

### Utilitarian analysis

Utilitarianism claims that an act is morally right if and only if it maximizes the good or utility for the largest number of people. (Driver, 2014; Marseille and Kahn, 2019). Utilitarianism is not focused on to whom the benefits are distributed when utilizing a population genomic screen.

Health economics and outcomes research such as a cost-utility analysis can assist with providing insight into what actions maximize benefits for a population. (Beheshti et al., 2018). For this utilitarian analysis, we focused on the clinical implications of screening along with the clinical benefits and risks related to surveillance, preventative therapeutics, or interventions, and/or the need for surgical prophylaxis. Clinical benefits and risks were contextualized within the age of onset for disease.

FH is a condition with an “early” age of onset insofar as the adverse effects of increased cholesterol levels begin prior to the experience of a cardiovascular event such as MI or stroke. (Ademi et al., 2019). FH diagnosis does not have an associated prophylactic surgery that affects the risk level of affected individuals but does have therapeutic options. (Simon Broome Register Group, 1991; Newman et al., 2019). Surveillance and preventative intervention may provide meaningful benefit through cholesterol screening, lipid lowering therapy, and related clinical actions. Research has shown that children undergoing population genetic screening is likely cost-effective and has benefit in a non-US setting. (Sturm et al., 2018; Ademi et al., 2020). Preliminary results from Spencer et al. indicate that population genomic screening is more cost-effective for younger patients (20-year-old compared to 35-year-olds). (National Cancer Institute, 2021). While there are potential side effects of lipid lowering therapy such as diabetes mellitus, and muscle pain or weakness, the overall safety profile of lipids suggests that they are relatively well tolerated by most patients. (Spencer et al.). As lowering patients’ cholesterol levels *via* statin use has high lifetime benefits and relatively low iatrogenic risks, screening at the “Age of Majority” is preferred when this condition alone is considered.

HBOC, in contrast, is generally characterized as having a later age of onset due to a 4% cumulative risk of experiencing breast cancer up to age 30. (Manickam et al., 2018). As a result, most individuals with *BRCA* mutations experience a breast cancer diagnosis after the age of 30 and prophylactic surgery is recommended afterwards due to its invasive and irreversible nature. (National Cancer Institute, 2021). HBOC recommendations also include increased surveillance for affected individuals such as mammography or MRI. While genetic testing for HBOC is recommended for women who have a family history or who have experienced triple-negative breast cancer before age 60, (Nelson et al., 2019) *Guzauskas et al.* found that population genomic screening for HBOC was likely cost-effective for 30-year-old women. (Guglielmo et al., 2018; Guzauskas et al., 2020). Due to the availability of effective surveillance, the majority of cancer diagnoses occurring after age 30, and the recommendation of prophylaxis after age 30, screening “After the Age of Majority” is preferred when this condition alone is considered.

The typical age of onset of LS is also variable; nevertheless those who screen positive for LS are recommended to pursue colonoscopy annually or biannually beginning at the age of 25 or 25 years before the youngest familial CRC diagnosis, as well as to consider annual endometrial sampling or transvaginal ultrasound (TVUS) where relevant, and/or esophagogastroduodenoscopy (EGD) beginning at age 30. (Beauchamp and Childress, 2001; Vasen et al., 2013; Giardiello et al., 2014a; National Comprehensive Cancer Network, 2021). Polyps identified by screening can be resected to significantly lower the likelihood that a patient will experience a late-stage cancer diagnosis or unknown cases of cancer. (Cleveland Clinic, 2022; Katz et al., 2017). In some cases additional prophylactic surgery may be necessary, such as a colectomy, or an oophorectomy for patients affected by endometrial or ovarian cancers (recommended after childbearing has been completed). Given the availability of effective surveillance, the recommendation for screening activities to begin at age 25, and prophylactic interventions connected to surveillance, screening “After the Age of Majority” is preferred when this condition alone is considered.

In summary and when considered independently, a utilitarian analysis of–primarily clinical–implications suggests that it is more appropriate to offer screening for both HBOC and LS “After the Age of Majority” whereas screening for FH may be preferred at the “Age of Majority” as shown in Table 2. As a panel test, however, and under a “majority rules” understanding, on balance it would be better to offer a combined test “After the Age of Majority”. This recommendation, which could delay lipid lowering interventions for those with FH, nevertheless carries fewer risks than initiating expensive and (for LS, invasive) surveillance modalities well in advance of the expected age of disease onset.

**TABLE 2:** Utilitarian analysis.

Condition	Utilitarian recommendation	Rationale
FH	“Age of Majority”	- Availability of effective surveillance
		- Majority of cancer diagnoses occurring after age 30
		- Recommendation of prophylaxis after age 30
HBOC	“After Age of Majority”	- Availability of effective surveillance
		- The majority of cancer diagnoses occurring after age 30
		- Recommendation of prophylaxis after age 30
LS	“After Age of Majority”	- Availability of effective surveillance
		- Recommendation for screening activities to begin at age 25
		- Prophylactic interventions connected to surveillance
Narrow Panel Test	“After Age of Majority”	- Analysis recommends 2 of the 3 conditions at “After Age of Majority”

There are, as noted above, additional implications not easily integrated into these considerations. Building on the utilitarian analysis, the same case was evaluated using the ethical framework of principlism, with an additional focus on non-clinical and family planning implications.

### Principlist analysis

Principlism applies the ethical principles of respect for autonomy, justice, beneficence, and non-maleficence to consider the morality of an action. (Beauchamp and Childress, 2001; Pal and Vadaparampil, 2012). Respect for autonomy is an individual’s ability to make decisions for themselves with adequate information about the consequences of their choices and without coercion. Beneficence refers to acting to benefit others which may involve preventing harms or actively promoting some sort of specific benefit(s). Non-maleficence refers to not intentionally causing harm or avoiding actions that are expected to harm individuals. Justice refers to considerations related to the fair distribution of the benefits and harms or costs of an action. While joint consideration of these principles can often point to a consistent course of action, in practice different principles may lead to different evaluations of the morality of an action. Table 3 highlights which principles present discordance with the utilitarian recommendation.

**TABLE 3:** Principlist analysis.

	Respect for autonomy	Beneficence	Non-maleficence	Justice
FH	(+)	(-)	(+)	(+)
HBOC	(-)	(+)	(-)	(-)
LS	(-)	(+)	(-)	(+)

(+): Indicates discordance from Utilitarian Recommendation.

(-): Indicates no discordance from utilitarian recommendation.

Respect for autonomy is relevant to considerations surrounding family planning. Individuals may want to take steps to limit the likelihood of passing a risk variant to offspring *via* preimplantation genetic diagnosis or related activities. Having risk information at the “Age of Majority” may provide additional time for reproductive planning, allow affected individuals to stop or delay therapeutic interventions, such as statin therapy for FH, when intending to conceive a child, or to delay prophylactic interventions such as a mastectomy for HBOC. (Wert, 2005; McGowan et al., 2019). Implementing a narrow panel test “After the Age of Majority” therefore interferes with patients’ autonomy by limiting their ability to make such decisions in a timely fashion.

Waiting until “After the Age of Majority”, may raise issues with family members’ autonomy by not respecting their right not to know their own genetic status. (Koçan and Gürsoy, 2016). Similarly, not all individuals may benefit from implementation “After the Age of Majority,” i.e., the utilitarian recommendation, raising broader beneficence concerns. Individuals may be exposed costs or harms of unnecessary screening, especially since many patients will not receive a positive result, in contrast with providing benefits to the population at large. Providing opt-out options for patients who do not feel they will benefit may address this concern, in conjunction with educational resources regarding the purpose and potential benefits of such a program. Of course, autonomy may also be infringed by an earlier age of implementation, where strongly encouraged clinical actions, such as mastectomy in females, have noted to negative impacts on self-image, body image, identity, or other factors. (Kenen et al., 2007; Petrucelli et al., 2016).

Non-maleficence and beneficence may appear in discordance with one another. With an opt-out option for the narrow panel test, individuals may wish to opt-in to screening for a particular condition or disease prior to the recommended time. However, individual conditions within a panel test may challenge the timing of a screen in relation to doing no harm. An opt-in option, or adequate information and counseling for individuals who would elect to begin screening earlier than proposed, may help individuals and other stakeholders limit patient harm while allowing pragmatic implementation.

There is potential for undue harm from utilizing a narrow panel test at too young an age. The possibility of exposing individuals to information that leads to unnecessary prophylaxis such as a mastectomy, oophorectomy, or colectomy could cause undue harm. (Howard et al., 2010; Rendle et al., 2015). Risk reducing prophylaxis presents the potential for psychosocial harm. (Hamilton et al., 2017; Shugar, 2017). Non-maleficence may exist within a panel test as a result of these potential harms and is important to identify explicit trades-offs to limit harms. Clear training and provider familiarity with the clinical care pathways can assist with minimizing the risk of these harms. (Bensend et al., 2014). Provision of educational programs and access to genetic counseling can assist with balancing benefits against risks such as anxiety or psychosocial impacts. (Khera et al., 2016).

Justice considerations center on the degree to which specific subsets of (potentially already marginalized or underserved) patients may be unfairly impacted by the age of implementation chosen to maximize utility for the overall population. For example, with implementation at the “Age of Majority”, females may experience increased impact related to their family planning and non-clinical dimensions due prophylactic surgery such as mastectomy and/or oophorectomy. (MD Anderson Cancer Center, 2010; Collier, 2012; Centers for Disease Control and Prevention, 2020). Additionally, people who are pregnant or trying to conceive are not able to stay on statin therapy, increasing their cardiovascular risk. Earlier screening may give additional time to mitigate these potential harms and increase potential benefits through different family planning activities including when to attempt conception, how many children to have, and therapeutic interventions and conception(s) timing.

As with the application of the Utilitarian framework, competing considerations are in play when the principles are applied to implications associated with screening. Whereas a Utilitarian consideration suggests that implementation “After the Age of Majority” may, overall, be most appropriate, Principlism allows for broader consideration of implications. This additional consideration is important because the non-clinical or family planning implications, while more difficult to quantify, can be highly impactful as noted above. Principlist considerations do not change the over-arching conclusion that offering panel testing may be more appropriate at later life stages, but it *does* suggest important trade-offs with potential implications for responsible implementation. For example, offering population-based genomic screening on an opt-in basis, while desirable to respect patients’ autonomy, may expose patients to harms related to delayed diagnosis or put providers at risk of failing in their duty to do no harm. Similarly, implementing panel testing fairly may require restrictions related to individuals’ autonomy. While fairest to offer the screen to everyone at the same age, this may restrict the autonomy of those who wish to participate in screening earlier in adulthood.

It is important to realize that while this analysis assumed a population representative of the US population, this may not be the case for many health systems. Differences in disease prevalence by population background, or the presence of additional conditions, may need to be considered in relationship to the benefits expected from engaging in aggregate screening activities. As a result, it will be important to also consider appropriate demographic data when utilizing a principlist approach including non-clinical and family planning implications.

## Conclusion

For the hypothetical ‘narrow’ panel test considered here, our two-phase ethical analysis suggests that the most appropriate age of implementation may be “After the Age of Majority” (i.e., at 26 years of age or later). This conclusion is supported by the availability of cancer surveillance activities, recommendations for screening activities to begin at age 25, and prophylaxis to be considered after age 30 for HBOC and LS. While this timing is less optimal for FH screening, when considered as part of a panel test, our assessment is that the risks of delayed screening for FH are outweighed by other benefits. As we have demonstrated, a pragmatic approach can begin from a Utilitarian ethical framework based in a consideration of clinical implications, in a manner consistent with the need for health systems to weigh impacts on clinical outcomes relative to budgetary constraints, fiduciary responsibilities, and complex regulatory landscapes. Invoking Principlism in a secondary analysis considering non-clinical and family planning implications can then supplement the Utilitarian approach by identifying additional trade-offs.

## Future research

Future work in this space will assist with providing context for evaluations surrounding larger panel tests, which may include many more conditions than the current CDC Tier 1 genomic applications. These future analyses may encompass a broader set of potential implications including those associated with disease prevalence, modes of inheritance, and condition characteristics such as age of onset, severity, and other components. Explicit evaluation of non-clinical and family planning dimensions through discrete choice experiments or other qualitative and quantitative methods would add more insight into areas of ethical discordance. This work may allow for more accurate assessment of individuals’ preferences providing more appropriate and thorough considerations of the age at which panel testing should be implemented.
